# Novel distillation process for effective and stable separation of high-concentration acetone–butanol–ethanol mixture from fermentation–pervaporation integration process

**DOI:** 10.1186/s13068-018-1284-8

**Published:** 2018-10-20

**Authors:** Huidong Chen, Di Cai, Changjing Chen, Jianhong Wang, Peiyong Qin, Tianwei Tan

**Affiliations:** 1 College of Chemical Engineering, Beijing University of Chemical Technology, Beijing, 100029 People’s Republic of China; 2 Center for Process Simulation & Optimization, Beijing University of Chemical Technology, No. 15 Beisanhuan East Road, Chaoyang District, Beijing, 100029 People’s Republic of China; 3 National Energy R&D Center for Biorefinery, Beijing University of Chemical Technology, No. 15 Beisanhuan East Road, Chaoyang District, Beijing, 100029 People’s Republic of China

**Keywords:** Distillation, Process optimization, Acetone–butanol–ethanol separation, Downstream processing

## Abstract

**Background:**

One of the major obstacles of acetone–butanol–ethanol (ABE) fermentation from renewable biomass resources is the energy-intensive separation process. To decrease the energy demand of the ABE downstream separation processes, hybrid in situ separation system with conventional distillation is recognized as an effective method. However, in the distillation processes, the high reflux ratio of the ethanol column and the accumulation of ethanol on top of the water and butanol columns led to poor controllability and high operation cost of the distillations. In this study, vacuum distillation process which is based on a decanter-assisted ethanol–butanol–water recycle loop named E-TCD sequence was developed to improve the conventional separation sequence for ABE separation. The permeate of in situ pervaporation system was used as the feed.

**Results:**

The distillation processes were simulated and optimized by iterative strategies. ABE mixture with acetone, butanol and ethanol concentrations of 115.8 g/L, 191.4 g/L and 17.8 g/L (the other composition was water) that obtained from fermentation–pervaporation integration process was used as the feed. A plant scaled to 1025 kg/h of ABE mixture was performed, and the product purities were 100 wt% of butanol, 99.7 wt% of acetone and 95 wt% of ethanol, respectively. Results showed that only 5.3 MJ/kg (of butanol) was required for ABE separation, which was only 37.54% of the energy cost in conventional distillation processes.

**Conclusions:**

Compared with the drawbacks of ethanol accumulation in butanol–water recycle loop and the extremely high recovery rate of ethanol in conventional distillation processes, simulation results obtained in the current work avoided the accumulation of ethanol based on the novel E-TCD sequence.

**Electronic supplementary material:**

The online version of this article (10.1186/s13068-018-1284-8) contains supplementary material, which is available to authorized users.

## Background

Biobutanol (*n*-butanol) is an attractive alternative fuel and an important bulk chemical [1]. The production of biobutanol by acetone–butanol–ethanol (ABE) fermentation processes (mainly using Clostridia strains) from biomass materials is encouraged by government [2]. However, the bottleneck of low solvents concentration, yield and productivity which were caused by severe end-product inhibition, resulted in an economically unsatisfactory outcome that the ABE fermentation process can not be widely applied [3]. One way to solve the product toxicity is to improve the tolerance and solvents production of the Clostridia by revolution engineering and genetic methods [4, 5]. However, low butanol concentration was always obtained in fermentation broth, resulting in an energy-intensive downstream separation process [6, 7]. It is estimated that energy cost was the second highest production cost (occupying 14%) of biological ABE [8].

Another problem solution is coupled fermentation with in situ product recovery (ISPR) [9]. By the effect of ISPR, the productivity, the concentration and the yield of ABE were improved significantly [8, 10]. Among a wide range of ISPR techniques that have been coupled with ABE fermentation, pervaporation showed promising high separation efficiency with no harm to the culture [11]. Moreover, pervaporation is not limited by the vapor–liquid equilibrium; thus, higher titer production could be generated. Hence, the downstream energy demand might be further decreased [12, 13].

Nevertheless, in terms of industrialization, the commercial guide ABE production could not be obtained by solely practicing ISPR [14, 15]. A subsequent distillation system is still necessary for solvents purification [8, 16, 17]. Compared to the energy-intensive distillation that directly fed with the ABE fermentation broth, separation processes that coupled ISPR techniques with distillation could also significantly reduce the energy demand in ABE processes [11, 18, 19]. Lately, novel downstream processes such as vacuum separation–distillation [20], gas stripping–distillation [13, 19], extraction–distillation [21–23], pervaporation–distillation [11, 24] and the hybrid separations–distillation were well developed and deeply analyzed [13].

**Fig. 1:**
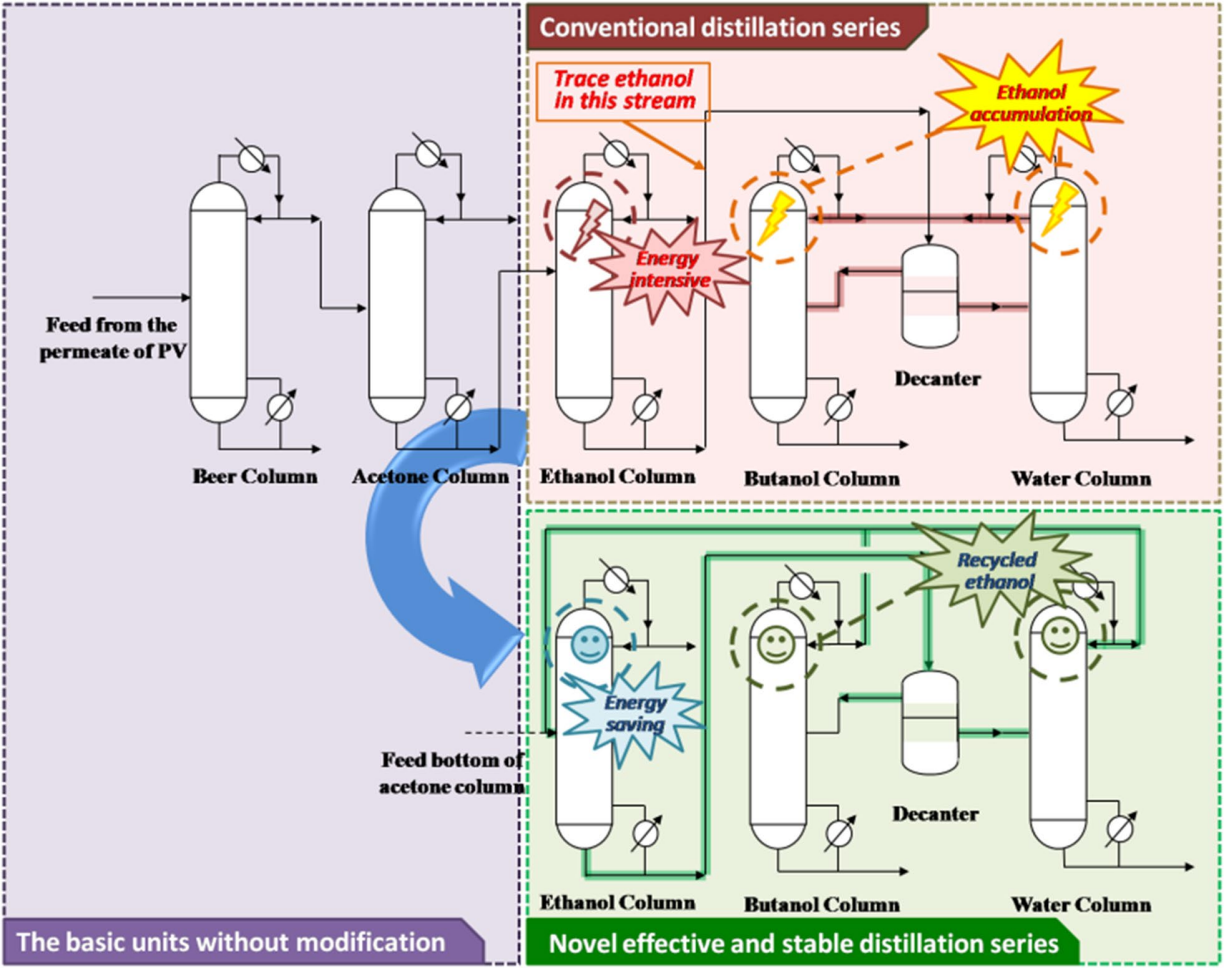
Differences between the conventional distillation series and the novel distillation series. In the conventional distillation series, the energy requirement of the ethanol column was high. There was a trace of ethanol contained in the bottom stream of ethanol column, which would further accumulate in the distillates of water and butanol columns. The process is poor in controllability in long-term operation. The novel effective and stable distillation series only changed the streams of the water and butanol columns’ distillates. The reflux ratio of the ethanol column decreased and no ethanol accumulated in the water and butanol columns

Typically, the ABE fractions after ISPR were commonly separated in sequence of the boiling points in the distillation units (Fig. 1). In the final step, water–butanol mixture was separated by ‘two columns + decanter’ (TCD) system [25]. However, the high reflux ratio of the ethanol column caused by the low titer ethanol concentration in feed would hugely increase the heat demand. In addition, ethanol, the lighter component, might be accumulated on top of water and butanol columns. As it was mentioned in Patraşcu et al. [19] study, the conventional distillation series showed in Fig. 1 was of poor controllability, whilst the operation cost was also high. Although the extraction–distillation system might help to solve this problem [21, 23], the extractive recovery was energy-intensive and environmentally unfriendly. In contrast, obtaining a phase-separated ABE mixture was another feasible method [19]. Unfortunately, a phase separation mixture by ISPR was not universally applied. For example, because the acetone separation efficiency is higher than that of butanol and ethanol, the permeate of pervaporation by ISPR was always rich in acetone fraction, which further led to a homogenous phase in the feed stream of distillation [10, 12, 26].

Thus, an effective and stable distillation separation process should be developed to meet the demands of the subsequent ABE distillation separation after ISPR. In the present study, the conventional distillation series was improved by recycling the distillate of the water and butanol columns to the ethanol column. The results were beneficial to the downstream processes because not only the heat demand of the ethanol column decreased, but also the problem of ethanol accumulation in the TCD system was solved. The novel distillation process was further optimized by increase/decrease of column pressure and pinch analyses.

## Methods

### Upstream ABE fermentation–pervaporation integration process

The feeding stream of ABE in distillation unit was the permeate of pervaporation system which was obtained from the upstream ISPR process. The fermentation–pervaporation integration process was laboratory operated, which was similar to our previous reports using sweet sorghum juice as the substrate [10, 12]. Diluted sweet sorghum juice that contains 72.1 g/L of total fermentable sugars (includes glucose, fructose and sucrose) was detected after inoculation of the seeds under a size of 10% (v/v). When the sugars in broth were almost used up, 8.5-fold concentrated sorghum juice was added into the bioreactor in fed-batch mode to ensure the sufficient carbon source and nutrient demands in ABE fermentation. The bioreactor was connected with a membrane module equipped with hydrophobic polydimethylsiloxane/polyvinylidene fluoride (PDMS/PVDF) membrane. During the process, the fermentation broth was recycled and accessed through the membrane module. On the permeate side of membrane, vacuum environment was developed. ABE condensate in permeate was attempted to be pumped into the distillation system (for details see Additional file 1: Fig. S1).

ABE concentrations were maintained at 15.8–18.4 g/L in fermentation broth. Correspondingly, the permeate was obtained with a stable ABE concentration ranging from 297.9 to 344.5 g/L after condensation. The average ethanol, acetone and butanol concentrations of 17.8 g/L, 115.8 g/L and 191.4 g/L were detected, respectively. Although it is evident that the ABE solution with more than 70 g/L of butanol is critical for phase separation [27], the ABE mixture obtained in the current work for downstream distillation separation is a monophasic solution without phase separation, which coincided with the results obtained in our previous works [28–30]. This phenomenon could be explained by the high ratio of acetone, the co-product in ABE permeate. Hence, the higher percentage of acetone in ABE mixture after pervaporation was proved as an adverse factor for phase separation [26]. In the simulation step, the monophasic ABE permeate was fed into the distillation system. Time course of solvent concentration, flux and separation factor of pervaporation were showed in Additional file 1: Fig. S1. Production–separation equilibrium was achieved in the integration process [31].

### Problem statement

The conventional distillation series showed in Fig. 1 was previously reported [13]. The distillation series is based on the different boiling point of ABE solvents. The permeate of pervaporation was first concentrated. Then, the solvent produced are separated one by one from the slightest component to the heaviest one. Basically, the drawbacks of the conventional distillation process were as follows:

- The bottom stream of the ethanol column contained trace of ethanol (e.g., according to our previous report, it estimated ~ 245 ppm) [13]. After feeding this stream into the TCD sequence, ethanol, the slighter component (compared to butanol and water) could not be separated from the bottom outlet streams of the water and butanol columns. Hence, ethanol would gradually accumulate in the TCD sequence in long-term operations, and finally influence the butanol purity and the separation efficiency [19]. Thus, it is necessary to redesign the recycle loop form to eliminate the influence of the accumulated ethanol by-products in TCD sequence.
- When atmospheric distillation columns were applied for ABE separation, the temperatures of the distillation streams were always close. Hence, there were no enough temperature differences for heat exchange. In contrast, by applying vacuum distillation processes (VDP), the boiling point of ABE might be changed, which could further result in larger differences of the streams’ temperature and contribute to decrease the energy requirement by heat exchange, as also indicated and performed in previous works [8].

### Strategies

To address the above problems, the following methods were used: recycling the distillates of butanol and water column into the ethanol column, and increasing/decreasing column pressure with parameters optimization.

### Recycling the distillates

A new recycle loop that mixes the distillates of water and butanol columns with the bottom stream of the acetone column was developed. The resulting stream was fed into the ethanol column. Key parameters including the flow rates of the distillates of water and butanol columns were optimized. The outlet butanol stream might be contaminated by a trace of ethanol by-product when the recycle flow rates were too low, while the energy requirement was high on conditions of unsuitable high recycle flow rates.

### Decreasing the column pressure

By decreasing the column pressures, the differences of the streams’ temperature were enlarged. Hence, it would be easier to develop the heat integration process. When changing the pressure of the columns, the condenser temperature on the overhead of columns could not be lower than the room temperature. Additionally, the pressure of the columns is negatively correlated with the relative volatilities of the components. However, the influence of the relative volatilities on the separation performances of the individual columns might be quite different. Thus, the effect of the pressure on the separation performances of each column should be investigated firstly. Then, the pressure of sensitive columns could be decreased, and the insensitive ones were pressurized or kept under atmospheric pressure. The above strategies could be adopted because it can enlarge the temperature differences of streams and enhance the relative volatilities of the hard separated components. The reflux ratio of the columns might be lower, changing the distillation into a lower-energy cost process.

### Process optimization

**Fig. 2:**
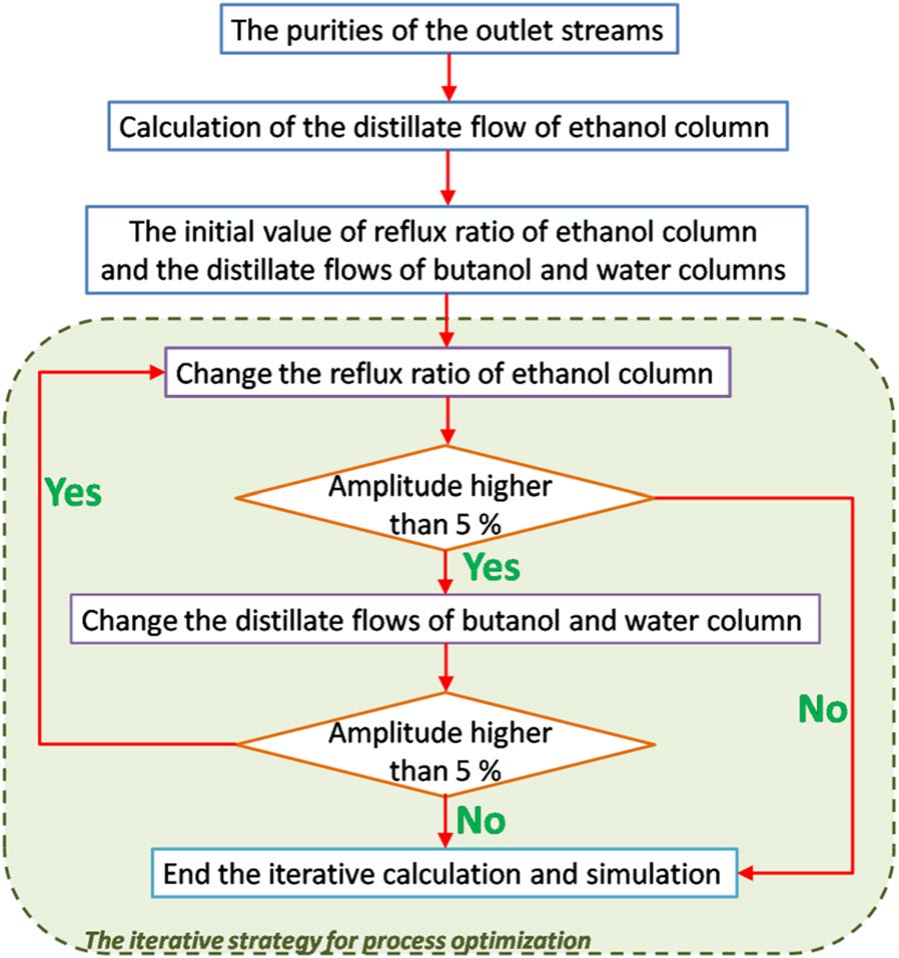
The sketch map of the logic in process simulation and optimization. In the recycled cycle of the novel distillation series (see green streams in Fig. 1), the reflux ratio of ethanol column and the distillate flows of the butanol and water columns were influenced by each other. An iterative strategy was developed to determine the parameters (inner the green boundary). The optimized condition was obtained by several iterations

The product purities of 95 wt% ethanol and anhydrous butanol and acetone (above 99.7 wt%) were the target of the distillation process which can meet the standard (GB/T 6026-1998). The processes were optimized by adjusting the recycled flow rates, the flow rates of distillate and bottom, and the reflux ratios. In the novel processes, streams were cycled in a system containing three columns (ethanol, water and butanol columns) and a decanter (E-TCD). The key parameters of the E-TCD sequences were influenced by each other. To optimize the novel E-TCD sequence, parameters were optimized by the following steps (also showed in Fig. 2):

- Calculating and determining the ethanol distillate flow to generate 95 wt% of ethanol product.
- Changing the distillate flow rates of the water and the butanol columns as well as the reflux ratio of ethanol column into the higher ones so that the purities of the output streams can be guaranteed.
- Gradually reducing the reflux ratio of the ethanol column and set it as low as possible.
- Gradually reduce the distillate flow rates of water and butanol columns under the promise to output suitable purities of solvents production. Minimizing the reflux ratio of the ethanol column can ensure the suitable purities of the output ethanol, butanol and water streams from their corresponding columns.
- Minimizing the distillate flows of the water and butanol columns. The regulation stopped when the changes were lower than 5%. Otherwise, go back to the first step and repeat the processes above.

### Heat exchange

Finally, based on the optimized conditions of the distillation streams, heat-exchange system was adopted to decrease the energy demand. The ultimate heating and cooling temperatures were set at 200 °C and 25 °C, respectively. Similar to our previous work [13], the minimum temperature difference for heat exchange was 15 °C.

To simplify the heat-exchange network, the following rules were applied:

- Heat exchange was terminated when the heat transfer rate was lower than 0.05 MJ/kg of butanol.
- Counter flow heat exchange was applied.
- Complete heat exchange was done in all of the streams, which means the difference of the two sides was 15 °C.

### Modeling approach

The distillation processes were simulated in UNISIM R380 software using NRTL property model which was used in previous references and suitable for ABE separation [32]. UNIQUAC model was used for the liquid–liquid equilibrium. Sequential Modular Approach was used in the model. The convergence tolerance was 1E−8. Two “convergence modules” were used to cut off the flows to ensure proper solution of the flowsheet applying a sequential modular simulation approach.

## Results and discussion

### Comparison of the TCD and E-TCD sequences based on atmospheric distillation

**Fig. 3:**
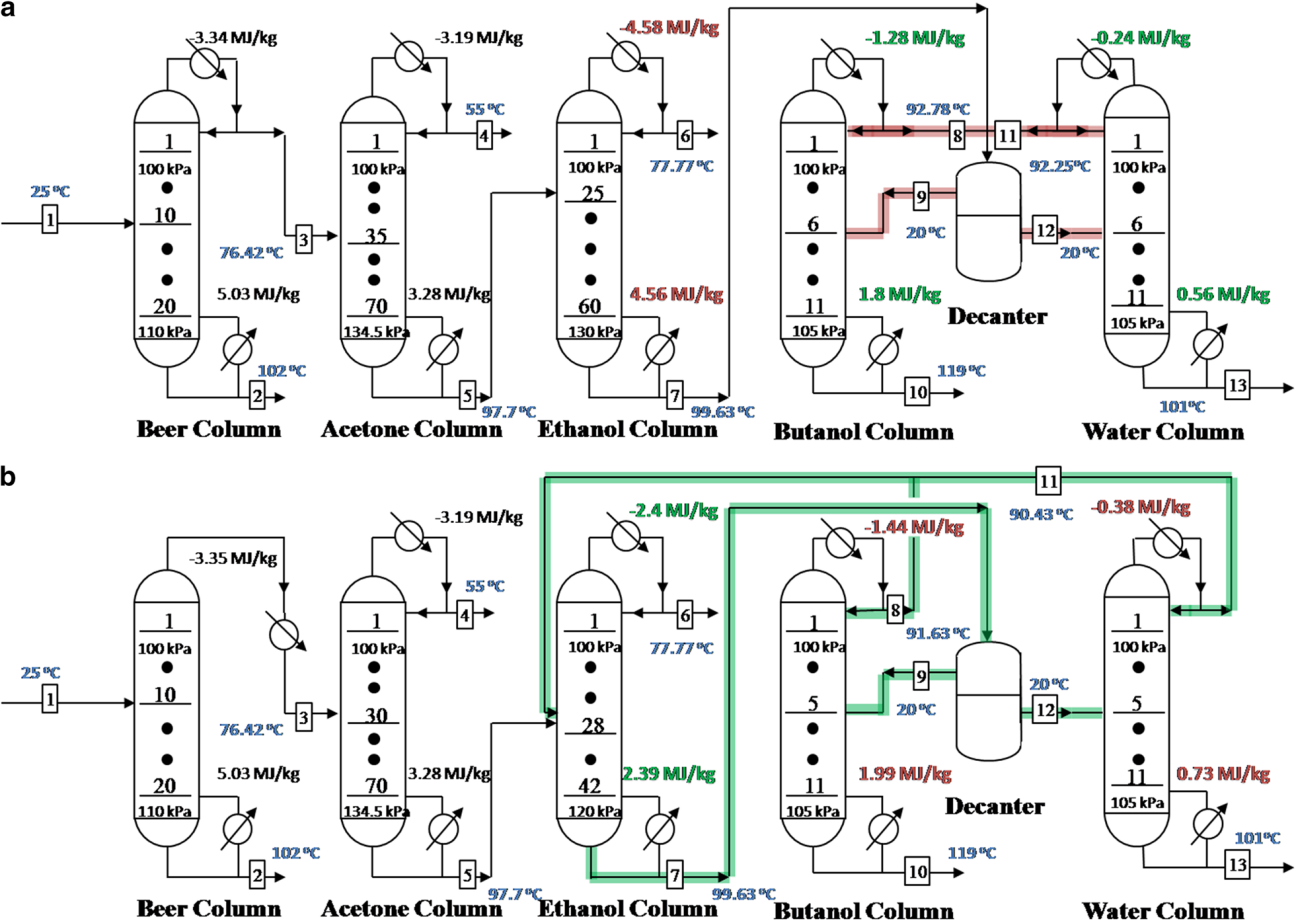
Atmospheric distillation processes representing **a** scenario 1 and **b** scenario 2. The flow rate in feed (stream 1) was 1025 kg/h. The red data are the higher-energy cost columns while the green data relatively required less energy in the corresponding columns. Black data refers to the heating and cooling energy that remained similar when recycling the water and butanol distillates. Feeding plates as well as the overall plates of each column were also shown in this figure. The overhead pressures of the columns were all set at atmospheric pressure

Atmospheric distillation processes which consisted of TCD (scenario 1) and E-TCD (scenario 2) sequences were developed and optimized firstly. Based on the construction and optimization strategies described in “Strategies” section, energy demands of the heating and cooling streams of the TCD and E-TCD sequences are illustrated in Fig. 3a and b, respectively. 95 wt% of ethanol (stream 6), 99.7 wt% of acetone (stream 4) and completely dehydrated butanol (100 wt%, stream 10) were obtained in both scenarios (detail stream composition and flows rate of different types of sequences are shown in Additional file 1: Table S1).

Data shown in Fig. 3 refer to the heating and cooling energy demand of distillation columns in two scenarios. As there were no differences between the upstream columns, the energy demands for the upfront two columns (beer and acetone columns) were also similar. Nevertheless, the results were quite different in TCD and E-TCD sequences in the downstream columns (ethanol, butanol and water columns). The lower energy requirement of the ethanol column in scenario 2 might be caused by the fact that a higher ethanol concentration in the bottom stream of the ethanol column is possible due to the recirculation loop. The stream which consisted of the distillate of butanol and water columns was mixed with the bottom outlet of the acetone column in scenario 2 (Fig. 2b). Thus, the actual flow rate that inlets into ethanol column in scenario 2 was 433.8 kg/h (the sum of flow rates from bottom outlet of acetone column and distillate of water column), which was higher than that of the case in scenario 1 (383.8 kg/h, only from the bottom outlet of acetone column). Nevertheless, the ethanol product flow rate in scenario 2 was not increased, which was maintained at 18.62 kg/h. Hence, the ethanol concentration of the bottom outlet of ethanol column in scenario 2 was higher than that in scenario 1 (2.42 wt% in scenario 2 vs. < 200 ppm in scenario 1), which could significantly reduce the energy requirement in ethanol column in scenario 2 (2.39 MJ/kg compared to 4.56 MJ/kg in scenario 1, see Fig. 3). Correspondingly, the reflux ratio of the ethanol column in scenario 2 (~ 25) was far lower than that in the scenario 1 (~ 57) when above 95 wt% of ethanol was reached (Additional file 1: Fig. S2).

Because of the lower concentrations of butanol in the organic phase from decanter (streams 9 in Additional file 1: Table S1) and the aqueous phase from decanter (streams 12 in Additional file 1: Table S1) in scenario 2 (81.66 wt% for the organic phase of the decanter and 4.6 wt% of the aqueous phase of the decanter), the heating and cooling energy requirement of the water and butanol columns was higher in the E-TCD sequence (1.99 MJ/kg and 0.73 MJ/kg for heating, and − 1.44 MJ/kg and − 0.38 MJ/kg for cooling in butanol and water columns, see Fig. 3b) in comparison to the conventional TCD sequence (1.8 MJ/kg and 0.56 MJ/kg for heating, and − 1.28 MJ/kg and − 0.24 MJ/kg for cooling in butanol and water columns, see Fig. 3a). Fortunately, the low butanol concentrations in both organic phase and aqueous phase in scenario 2 were mainly caused by the participation of the higher ratio of ethanol, which is the light component exist in the butanol–ethanol–water mixture [26]. Water fractions, the heavy component distributed in the mixture, did not increase significantly. Thus, the energy demand in water and butanol columns in scenario 2 were only slightly higher compared to the energy demand of the two columns in scenario 1.

Energy consumption in ethanol column was the decisive factor of the overall energy cost during the atmospheric distillations processes. Even though the energy cost for butanol and water columns was higher, the overall energy requirement for heating and cooling the streams in scenario 2 was far lower than that in the scenario 1. As a result, the energy demand of 13.42 MJ/kg and − 10.75 MJ/kg for heating and cooling, respectively, was consumed in scenario 2, which were only 88.1% and 85.1% of that compared to the energy demand in scenario 1. Therefore, the E-TCD sequence enables energy savings for the subsequent distillation separation ABE mixture after pervaporation. More importantly, as the ethanol (contained in the distillate of water and butanol columns) was recycled into the ethanol column in the E-TCD sequence, no ethanol accumulated in the TCD sequence (see Additional file 1: Table S1, streams 8 and 11). Hence, the E-TCD sequence showed a better controllability in contrast to the conventional TCD sequence. As it was suggested in previous report [19], the stable E-TCD sequence also enjoys the advantage of making the distillation system more cost effective.

**Fig. 4:**
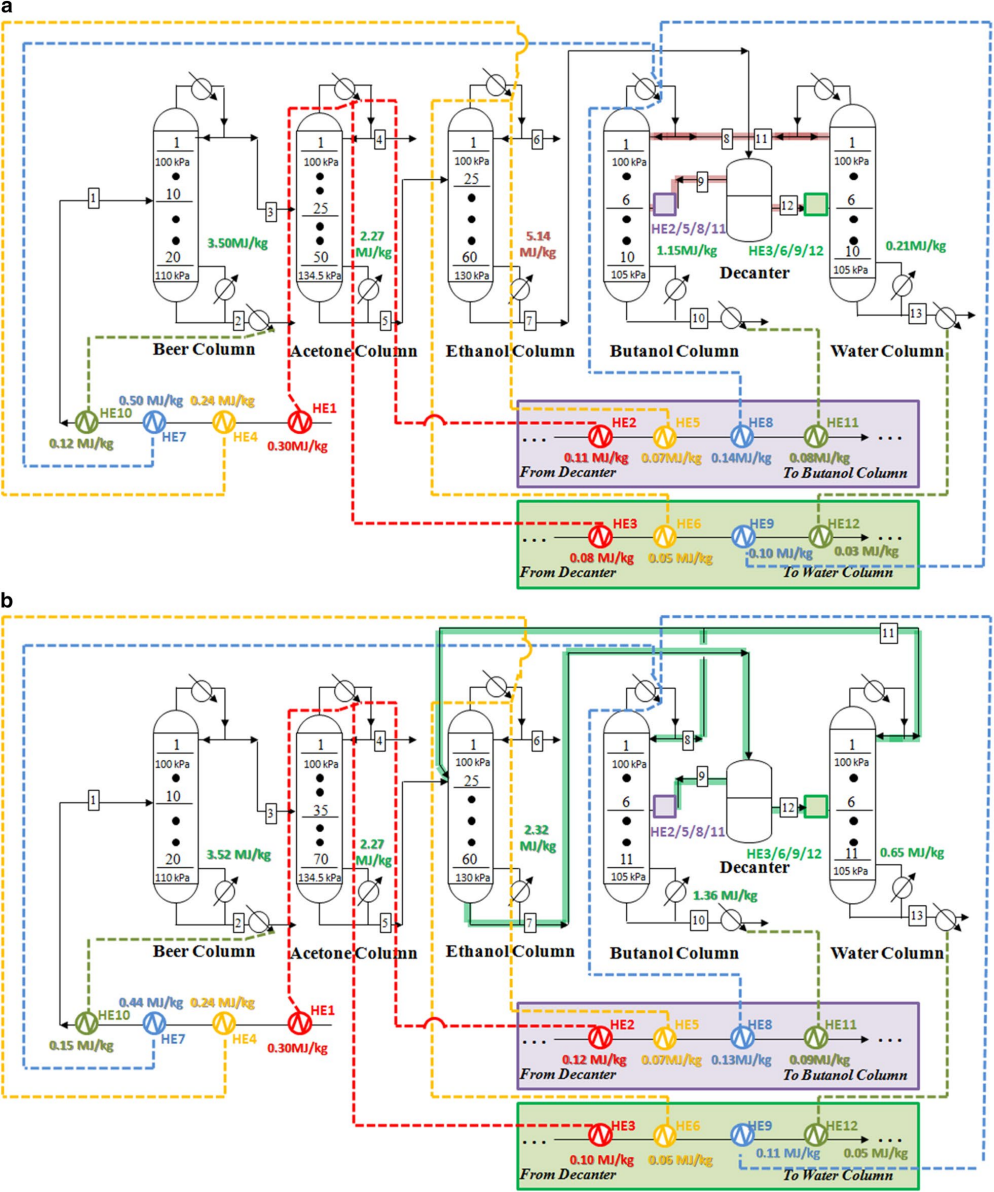
Heat-exchange system for the atmospheric distillation processes. **a** Heat-exchange strategies in scenario 1 which are based on TCD sequence; **b** based on E-TCD sequence

In our previous work, the application of heat-exchange system could significantly decrease the energy requirement in the conventional TCD sequence based on distillation process [13]. To further decrease the energy demands in the two scenarios, heat-exchange system was established and optimized. Energy requirements for ABE separation based on TCD and E-TCD sequences were also compared after the heat exchange. Generally, 12 heat exchangers (HEs) were connected with the streams in both scenarios. The minimum temperature difference for heat exchange was set at 15 °C. Key parameters and the heat-exchange strategies are carried out in Fig. 4. Besides, grand composites curves and the basic structure of the heat-exchange system are shown in Additional file 1: Fig. S3. As can be seen, under the optimized conditions, heat exchanges were mainly carried out in the streams 1, 9 and 12 in both scenarios, which were not in line with our previous works that applied the two-stage gas stripping–pervaporation process [13]. For the case of scenario 1, after heat exchange, the temperature of stream 1 gradually increased from 25 to 40.7 °C (after HE1), 62.77 °C (after HE4), 77.8 °C (after HE7) and 82.9 °C (after HE10), respectively. Similarly, the stream 9 which was fed into the butanol column was sequentially heated by HE2, HE5, HE8 and HE11, and its temperature finally reached 91 °C. Correspondingly, stream 12 (85.35 °C) was fed into the water column after being heated by HE3, HE6, HE9 and HE12. In contrast, in the case of scenario 2, the temperatures of stream 1, 9 and 12 were increased from 25 °C, 20 °C and 20 °C, to 84.25 °C (after HE1, HE4, HE7 and HE10), 91.6 °C (after HE2, HE5, HE8 and HE11) and 86 °C (after HE3, HE6, HE9 and HE12), respectively.

**Fig. 5:**
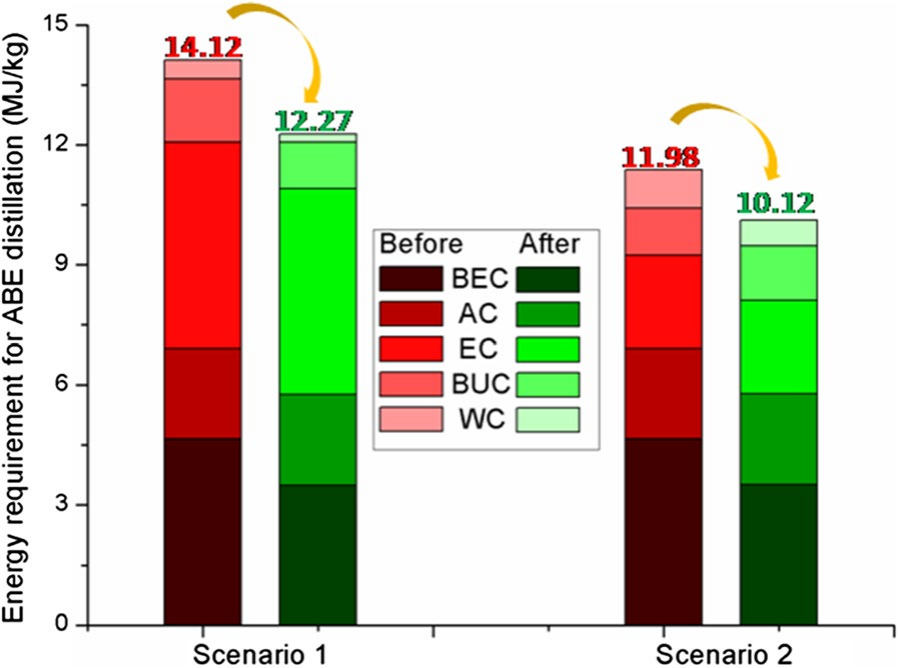
Comparison of the total energy requirements for the two scenarios of atmospheric distillation before and after the heat exchange

By the effect of heat exchange, the energy requirement of beer column, butanol column and water column were decreased. Energy requirements in both scenarios were decreased slightly. The energy demands of 12.27 MJ/kg and 10.12 MJ/kg were achieved in scenario 1 and scenario 2, respectively, which were 13.1% and 15.5% lower than that of the conventional processes without heat exchange (Fig. 5). Therefore, after heat exchange, energy requirement for the E-TCD sequence based on distillation (scenario 2) was still lower than that of the TCD sequence-based process (scenario 1), and the scenario 2 was more sensitive to heat integration for showing a relatively higher energy decreasing rate.

### The effect of column condenser pressures on the distillation performances and the improvement of the processes

Adjusting the pressure level of distillation columns showed advantages in further decreasing energy requirement in alcohols separation processes [32–35]. By applying VDP, the reflux ratios of several columns were decreased, and the heat exchange network was also intensified in VDP.

**Fig. 6:**
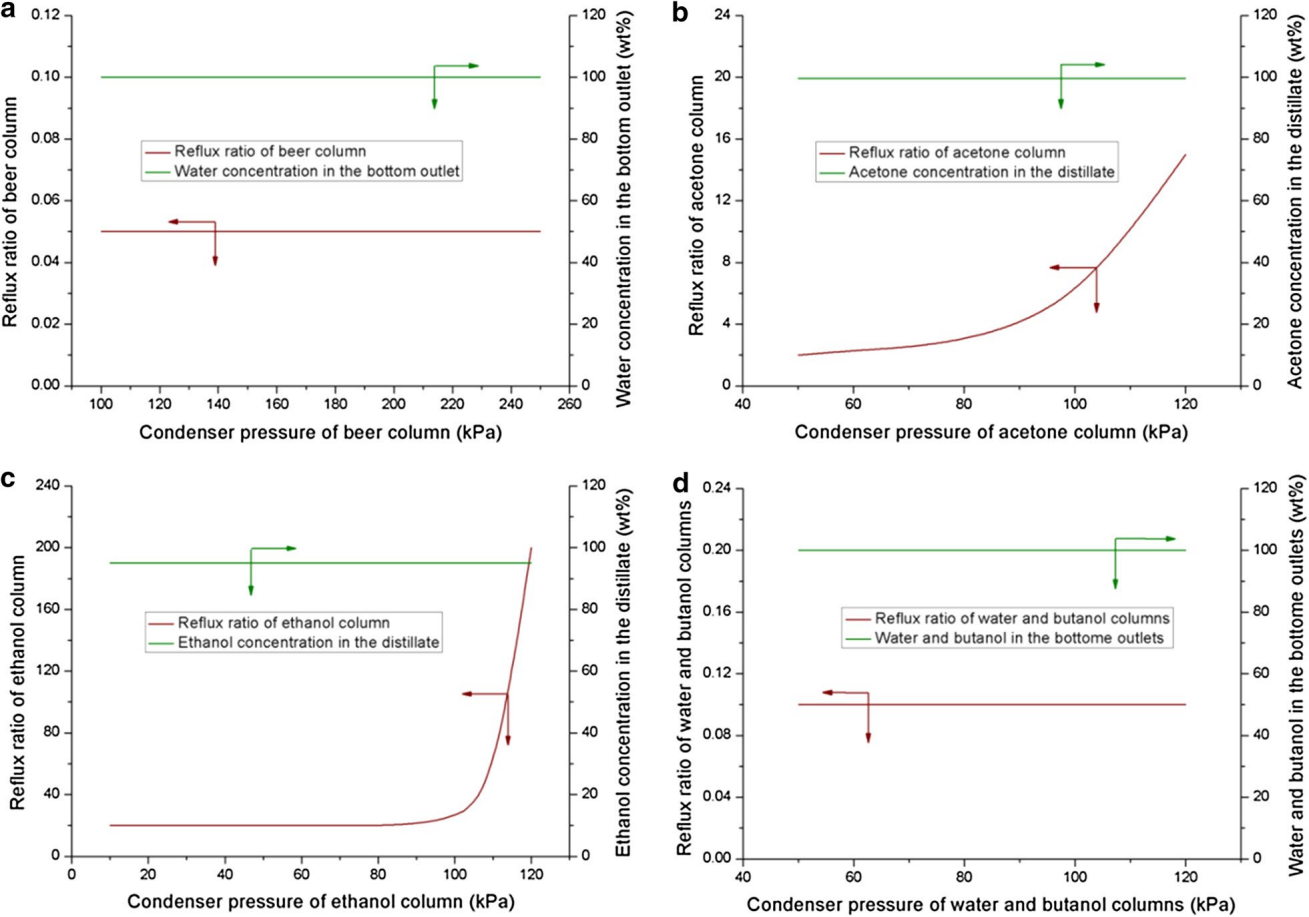
Effect of condenser pressure–reflux ratio in different distillation columns aiming to produce the acceptable products (95 wt% ethanol, 99.7 wt% acetone and 100 wt% butanol). **a** Beer column; **b** acetone column; **c** ethanol column; **d** butanol and water columns

In this section, VDP was applied for ABE separation based on E-TCD process. Figure 6 shows the effect of condenser pressures on the reflux ratios in output streams. In comparison to the beer, butanol and water columns, the reflux ratios of acetone and ethanol columns were more sensitive to the condenser pressures. To generate the acceptable acetone product in distillate, the reflux ratio of acetone column was gradually increased from 2 in 50 kPa to 15 in 120 kPa. By contrast, the reflux ratio of ethanol column did not change until the condenser pressure increased to 90 kPa. After that, the reflux ratio was significantly increased with the increase of condenser pressure, and finally reached 200 when the condenser pressure was 120 kPa. Hence, acetone and ethanol columns, the more sensitive ones, were selected to decrease the pressures.

The effect of the condenser pressures of acetone and ethanol columns on the distillate temperatures were evaluated. The distillate temperatures were decreased with the decrease of condenser pressures. 40 °C was considered to be the lowest temperature that can meet the needs of condensation (based on the minimum temperature for heat exchange of 15 °C). The suitable condenser pressures of acetone and ethanol columns were 57 kPa and 18 kPa, respectively (details are also shown in Additional file 1: Fig. S4).

After decreasing the acetone and ethanol column pressures to 57 kPa and 18 kPa, the effect of reflux ratios on the distillate acetone and ethanol concentrations was further investigated. The TCD (scenario 3) and E-TCD (scenario 4) sequences based on VDP were compared (details see Additional file 1: Fig. S5). After decreasing the condenser pressures of acetone and ethanol columns, the optimized reflux ratio for acceptable purities of solvents was decreased sharply in both of the TCD and E-TCD sequences Therefore, the energy consumption might be also decreased. More specifically, the optimized reflux ratio in acetone column was decreased from 5.8 to 2.4, while the optimized reflux ratio in ethanol columns was only 48 and 18 in the sequences of scenario 3 and scenario 4 after increasing/decreasing columns pressure, respectively.

**Fig. 7:**
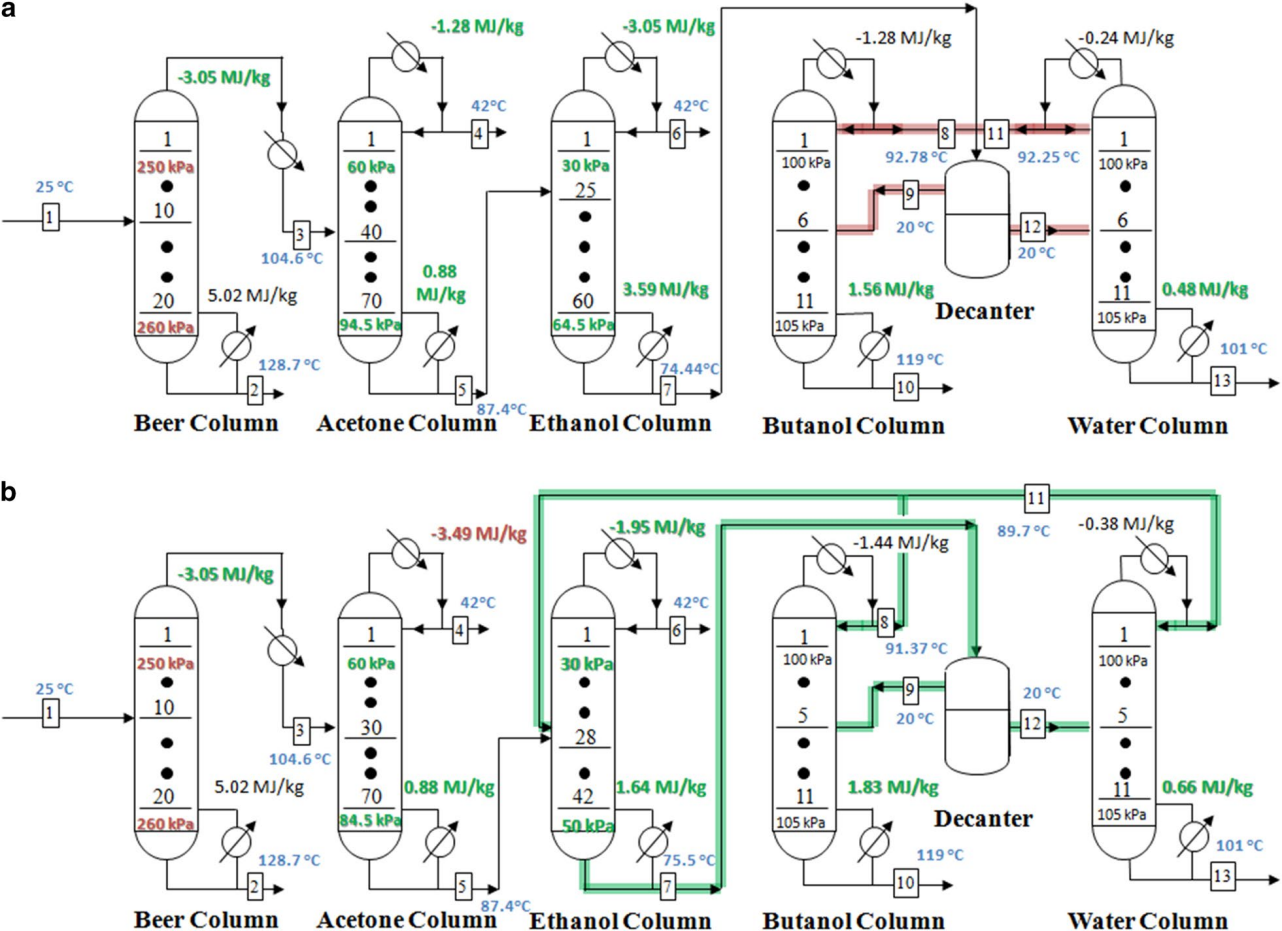
VDP representing **a** scenario 3 and **b** scenario 4. The flow rate in feed (stream 1) was 1025 kg/h. The red data are the columns with higher energy cost while the green data relatively requires less energy compared to the atmospheric distillations shown in Fig. 3. Feeding plates, overall plates as well as the overhead pressure of each column were also shown in this figure

After optimizing the condenser pressure of acetone and ethanol columns, key parameters of the water and butanol columns were subsequently determined by changing the distillate of butanol and water column following the iterative strategy shown in Fig. 2 (The stream flow rates showed in Additional file 1: Table S2). Based on the specific conditions for VDP, the key parameters of TCD and E-TCD sequences are conducted in Fig. 7. As it is illustrated, the condenser pressure of beer column was increased to make the process of transferring the heat easier. Accordingly, the pressures of the acetone and ethanol columns were decreased while the condenser pressures of butanol and water columns remained in the atmospheric pressure. It showed that the heat requirements for the acetone and the subsequent ethanol, butanol and water columns in the TCD and E-TCD sequences all decreased after adjusting the columns pressures. The overall energy requirements in scenario 3 and scenario 4 were 11.53 MJ/kg and 10.03 MJ/kg (Fig. 9), respectively, which were 81.66% and 83.72% compared with the energy requirement in scenario 1 and scenario 2 without applying VDP. Compared with other columns, the energy requirement for ABE concentration in beer columns occupied 43.54% (for scenario 3) and 50.05% (for scenario 4) of the overall heating cost. Additionally, although the heat requirement of the water and butanol columns in scenario 4 was a little higher than that of scenario 3, the sharp reduction of the energy cost in ethanol column also resulted in a lower overall energy demand in scenario 4.

**Fig. 8:**
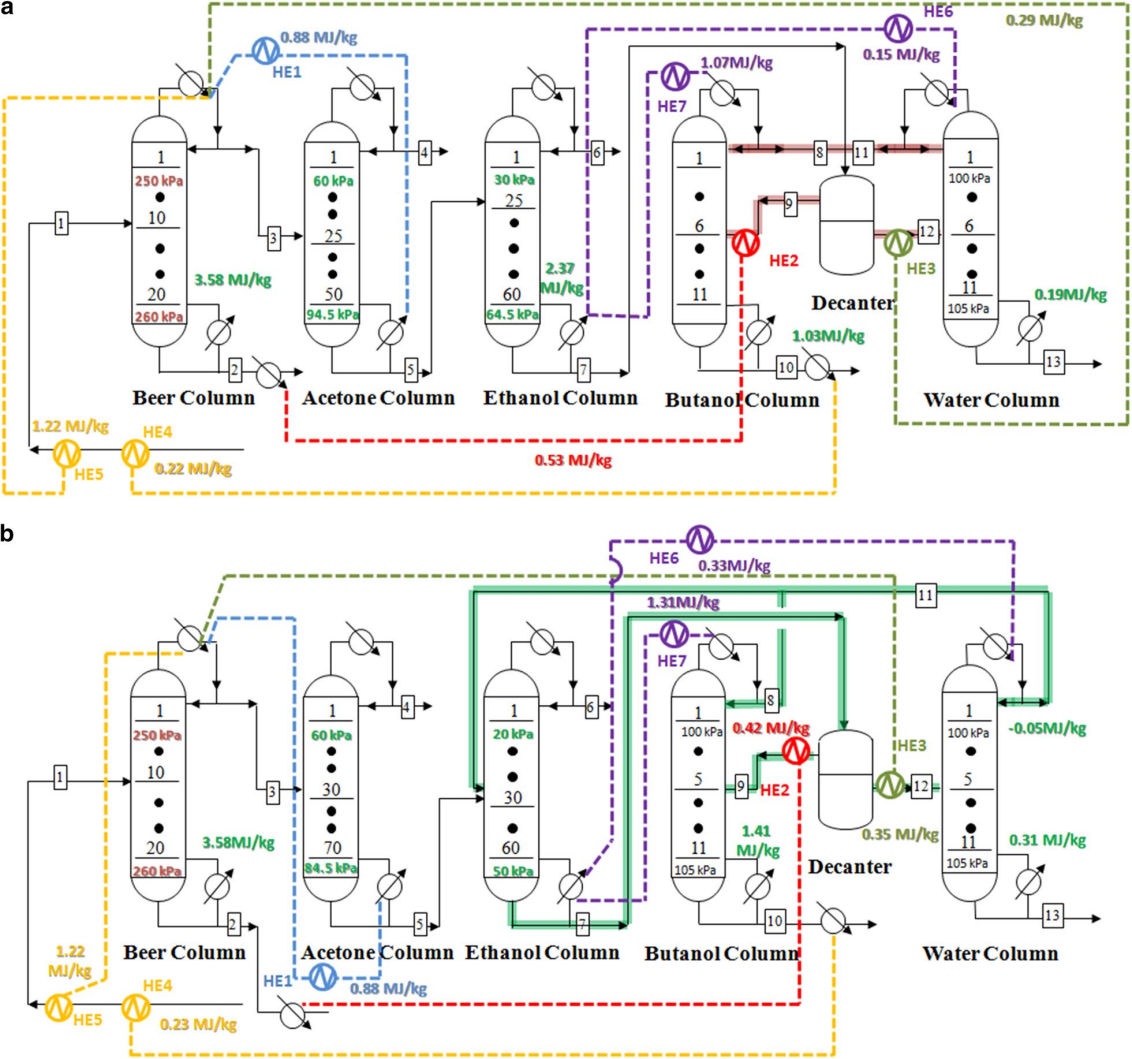
Heat-exchange system for VDP. **a** Heat-exchange strategies in scenario 3 which are based on TCD sequence; **b** and E-TCD sequence

**Fig. 9:**
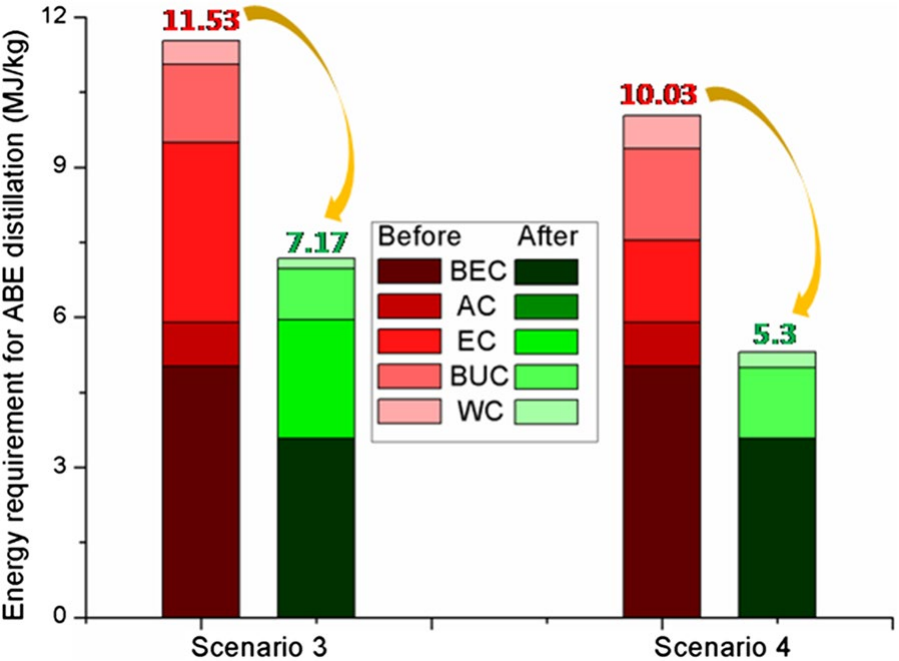
Comparison of the two scenarios of VDP in total energy requirements before and after heat exchange

Figure 8 shows the heat-exchange system for the VDP. Details of the grid diagram and grand composite curve were given in Additional file 1: Fig. S6. Compared to the VDP before heat integration, the energy requirement sharply decreased in both scenarios. Only 7.17 MJ/kg and 5.3 MJ/kg heat were consumed for ABE distillation separation from the permeate of in situ pervaporation separation in scenario 3 and scenario 4, respectively (Fig. 9). Under these conditions, 37.81% and 47.16% of energy could be saved after heat exchanges. Remarkably, it showed that no additional energy was required for heating the acetone and ethanol columns in scenario 4, and all the heat requirements were provided by the hotter streams. For the scenario 3, the bottom of acetone column was also warmed by the overhead product of beer column. It is also noteworthy that the number of heat exchangers can be, thus, reduced in scenarios 3 and 4 based on VDP (total 7 heat exchangers, see Fig. 8) compared with conventional distillations in scenarios 1 and 2 (total 12 heat exchangers, see Fig. 3).

## Conclusions

The novel E-TCD sequence in VDP proposed in the current work was promising in making ABE separation from the permeate of pervaporation efficient, which showed advantages including low energy demand, good stability and controllability. Compared to the conventional distillation processes, the major improvements were recycling the distillate of water and butanol columns back to the ethanol column. This improvement could not only decrease the high reflux ratio of ethanol column, but also solve the problem of ethanol accumulation on the head of water and butanol columns. Remarkably, the energy cost for ABE separation was only 5.3 MJ/kg of butanol. The novel process can be employed widely and makes the ABE fermentation processes more cost effective.

## Additional file

**Additional file 1: Fig. S1.** Fed-batch ABE fermentation integrated with pervaporation using sweet sorghum juice; **Fig. S2.** Influence of the reflux ratio of ethanol column on ethanol concentration in distillate. **Fig. S3.** Grid diagram, hot and cold composite, and grand composite curve of heat-exchange system for the atmospheric distillation processes. **Fig. S4.** Effect of condenser pressure of acetone and ethanol columns on the distillate temperatures. **Fig. S5.** Effect of reflux ratio of columns on the output purities of solvents production. **Fig. S6.** Grid diagram, hot and cold composite, and grand composite curve of heat-exchange system for the VPD. **Table S1.** Comparison of streams and flow rates of the TCD (scenario 1) and E-TCD (scenario 2) sequences based on atmospheric distillations. **Table S2.** Comparison of streams and flow rates of the TCD (scenario 3) and E-TCD (scenario 4) sequences based on VDP.

## Abbreviations

ABE: acetone–butanol–ethanol
VDP: vacuum distillation process
TCD: two columns + decanter
E-TCD: ethanol column-two (water and butanol) columns + decanter
ISPR: in situ product recovery
PDMS/PVDF: polydimethylsiloxane/polyvinylidene fluoride
NRTL: non-random two liquid
HE: heat exchanger
BEC: beer column
AC: acetone column
EC: ethanol column
BUC: butanol column
WC: water column
