# Supplementary material for: Novel distillation process for effective and stable separation of high-concentration acetone–butanol–ethanol mixture from fermentation–pervaporation integration process
Source: Biotechnol Biofuels. 2018 Oct 20;11:286. doi: 10.1186/s13068-018-1284-8 (PMC6195753; doi:10.1186/s13068-018-1284-8)
Supplement: Supplementary file 1 — Additional file 1: Fig. S1. Fed-batch ABE fermentation integrated with pervaporation using sweet sorghum juice; Fig. S2. Influence of the reflux ratio of ethanol column on ethanol concentration in distillate. Fig. S3. Grid diagram, hot and cold composite, and grand composite curve of heat-exchange system for the atmospheric distillation processes. Fig. S4. Effect of condenser pressure of acetone and ethanol columns on the distillate temperatures. Fig. S5. Effect of reflux ratio of columns on the output purities of solvents production. Fig. S6. Grid diagram, hot and cold composite, and grand composite curve of heat-exchange system for the VPD. Table S1. Comparison of streams and flow rates of the TCD (scenario 1) and E-TCD (scenario 2) sequences based on atmospheric distillations. Table S2. Comparison of streams and flow rates of the TCD (scenario 3) and E-TCD (scenario 4) sequences based on VDP. [file 13068_2018_1284_MOESM1_ESM.doc]

**Additional files**

Novel distillation process for effective and stable separation of high concentration acetone-butanol ethanol mixture from fermentation-pervaporation integration process

Huidong Chen ^a,b^, Di Cai^c*^, Changjing Chen ^c^, Jianhong Wang ^a,b*^, Peiyong Qin ^c*^, Tianwei Tan ^c^

^a^ College of Chemical Engineering, Beijing University of Chemical Technology, Beijing 100029, PR China

^b^ Center for Process Simulation & Optimization, Beijing University of Chemical Technology, Beijing 100029, PR China

^c^ National Energy R&D Center for Biorefinery, Beijing University of Chemical Technology, Beijing 100029, PR China

^*^ Corresponding authors

Address: No.15 Beisanhuan East Road, Chaoyang District, Beijing, 100029.

Email: caidibuct@163.com; wangjh@mail.buct.edu.cn; qinpeiyong@tsinghua.org.cn

Fig.S1 Fed-batch ABE fermentation integrated with pervaporation using sweet sorghum juice (a) Residual sugars concentration in bioreactor and the cumulated ABE production; (b) ABE and organic acids concentration remained in the bioreactor; (c) ABE concentration on the permeate side of pervaporation membrane (d) Kinetics of water and ABE flux with fermentation time; and (e) Kinetics of ABE separation factors with fermentation time.


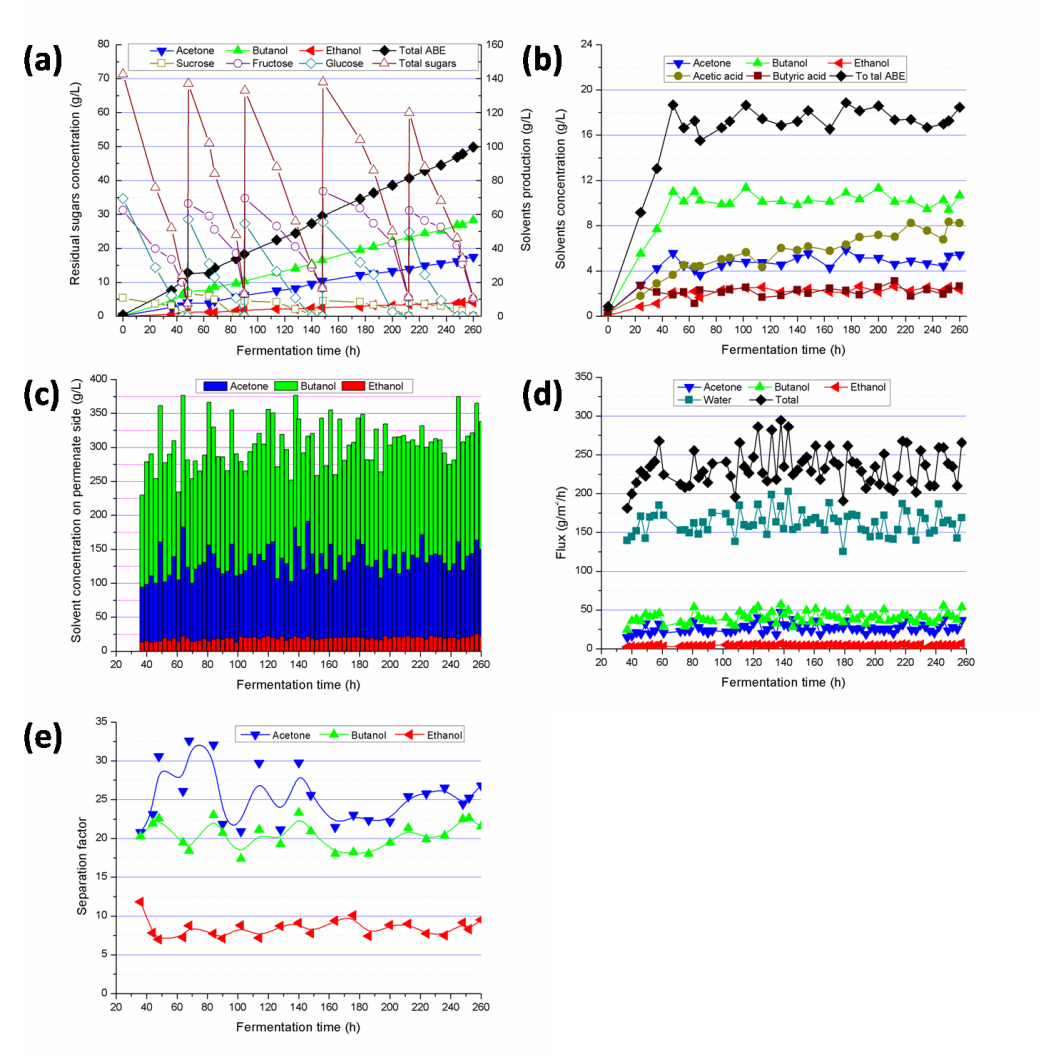


Discussion of Fig. S1:

As it showed in Fig. S1, after 260 h of fermentation and pervaporation, total 295 g/L of the fermentable sugars from concentrated sweet sorghum juice was converted into ABE production (Fig.S1a). In comparison with the stable ABE concentration remained in the fermentation broth (ABE concentration in bioreactor was ranged from 15.5 g/L to 19.0 g/L), the organic acid by-products, include acetic acid and butyric acid, were gradually increased and finally reached 8.2 g/L and 2.6 g/L, respectively (Fig.S1b). In the end of fermentation, acetone, butanol and ethanol concentrations remained in the fermentation broth were 5.4 g/L, 10.7 g/L and 2.4 g/L, respectively. Correspondingly, on the permeate side of membrane, concentrations of acetone, butanol and ethanol were fluctuated from 81.1 g/L, 125.4 g/L and 13.0 g/L to 169.5 g/L, 233.6 g/L and 25.4 g/L, respectively (Fig.S1c).

During the fermentation and pervaporation, the flux of acetone, butanol and ethanol were maintained at 17.4~38.1g/m^2^h, 22.7~55.7 g/m^2^h, and 2.8~5.9 g/m^2^h, respectively (Fig.S1d), while the separation factors of acetone, butanol and ethanol were maintained at 21.2~33, 17.5~23.4, and 6.7~12.5, respectively (Fig.S1e).

Fig.S2 Influence of the reflux ratio of ethanol column on ethanol concentration in distillate. Processes were simulated in the atmospheric conditions. The minimized reflux ratio of scenario 2 to the desired ethanol concentration was far below the scenario 1. Thus, the energy requirement of the ethanol column was relatively low in scenario 2. The red and green dotted line referred to the reflux ratio of ethanol column when the ethanol purity reached 95 wt. %.


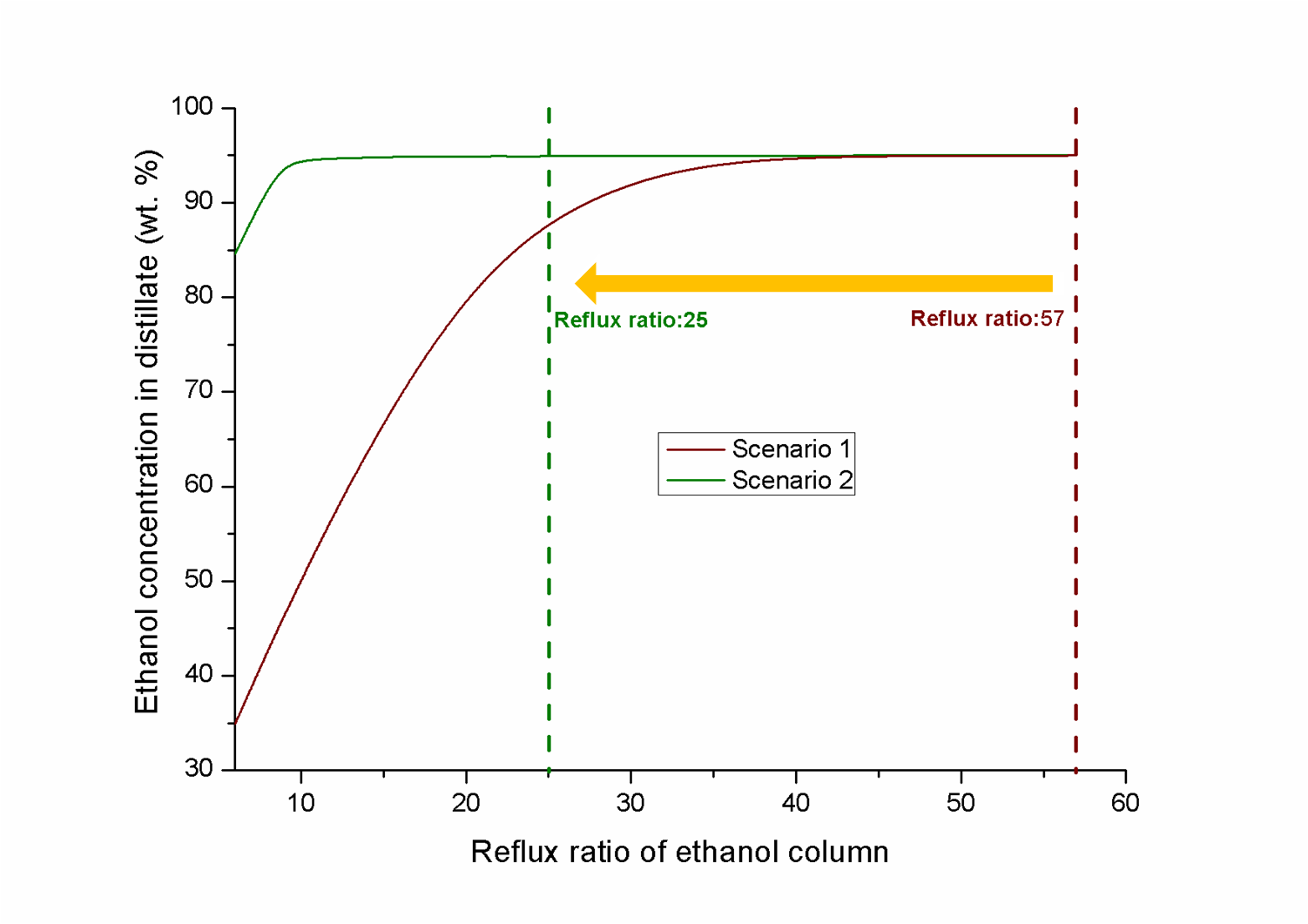


Fig.S3 Heat exchange system for the atmospheric distillation processes. (a) Grid diagram; (b) Hot and cold composite curve; and (c) Grand composite curve in scenario 1; (d) Grid diagram; (e) Hot and cold composite curve; and (f) Grand composite curve in scenario 2.


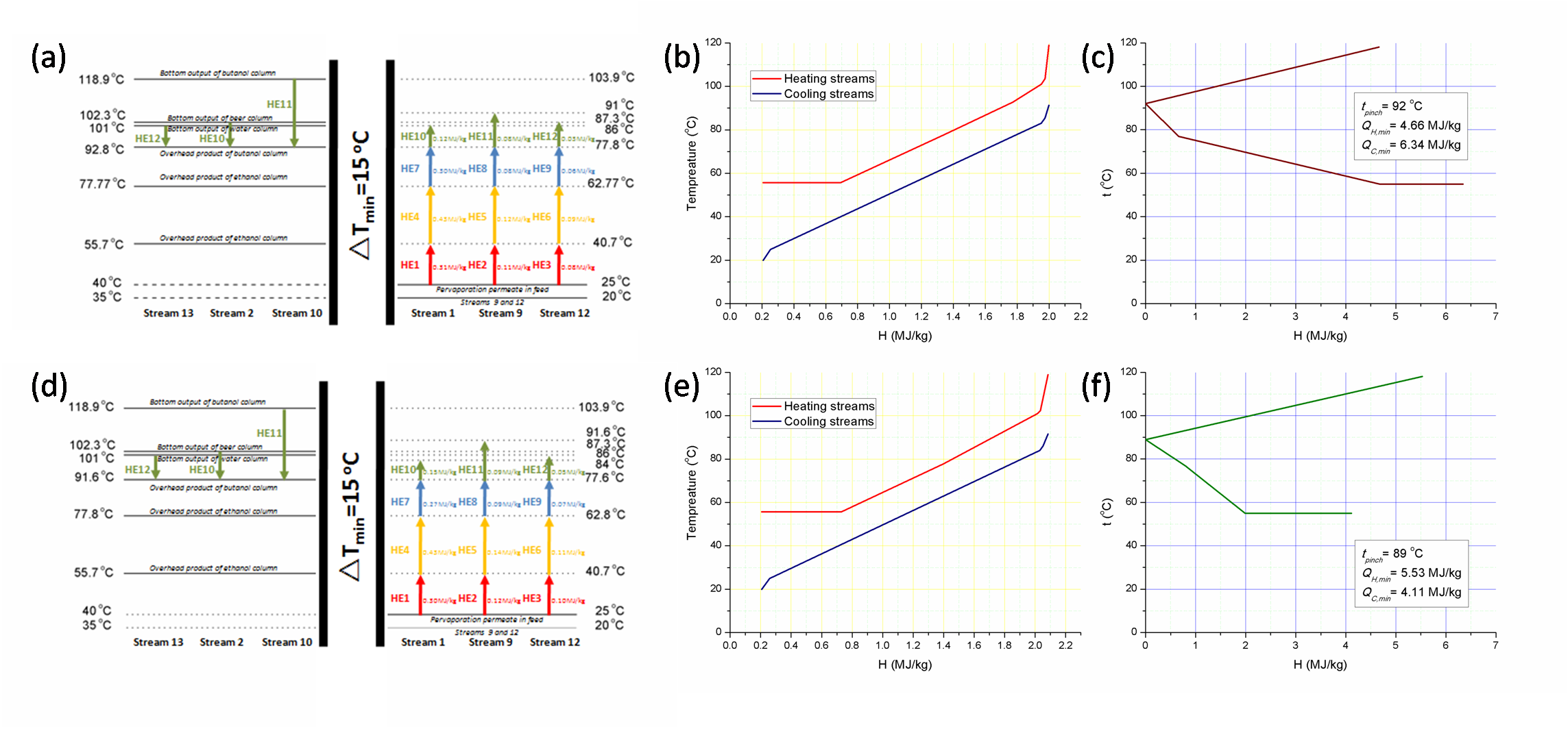


Fig.S4 Effect of condenser pressure of acetone and ethanol columns to the distillate temperatures. (a) Acetone column; (b) Ethanol column. The dotted line referred to the distillate temperatures, also the minimized cooling temperature of the top of column, when the temperature difference of the heat transfer was15 ^o^C and the cooling water temperature was 25 ^o^C. If the pressure on the head of column was lower than that of intersect of the dotted and solid lines, the cooling water temperature at 25 ^o^C could not be able to condensate the vapor in the column head.


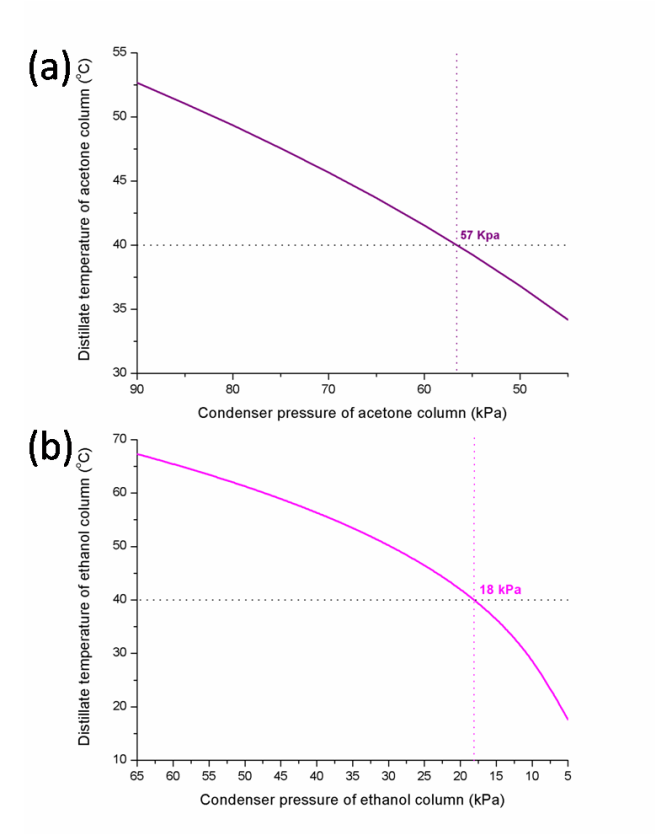


Fig.S5 Effect of reflux ratio of columns on the output purities of solvents production. (a) The acetone column. The green dotted line referred to the reflux ratio of acetone column when the purity of acetone product was higher than 99.7 wt. %. The distillate obtained in scenario 1 was similar to the scenario 2. Correspondingly, the scenario 3 was also similar with the scenario 4. It was because the acetone column was in front of the TCD and E-TCD sequences, and there were no obvious influence of the following sequences on the separation efficiency of acetone column. (b) The ethanol column. The red and green dotted lines referred to the reflux ratio of ethanol column when the purity of ethanol product was higher than 95 wt. %


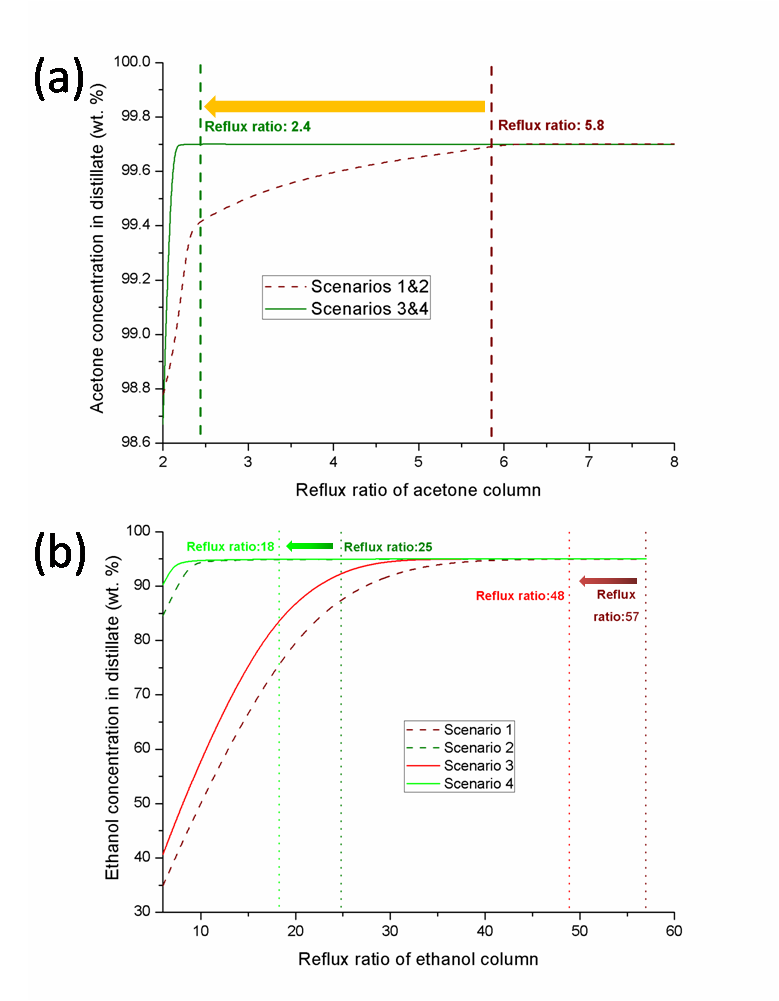


Fig.S6 Heat exchange system for the VDP. (a) Grid diagram; (b) Hot and cold composite curve; and (c) Grand composite curve in scenario 3; (d) Grid diagram; (e) Hot and cold composite curve; and (f) Grand composite curve in scenario 4.


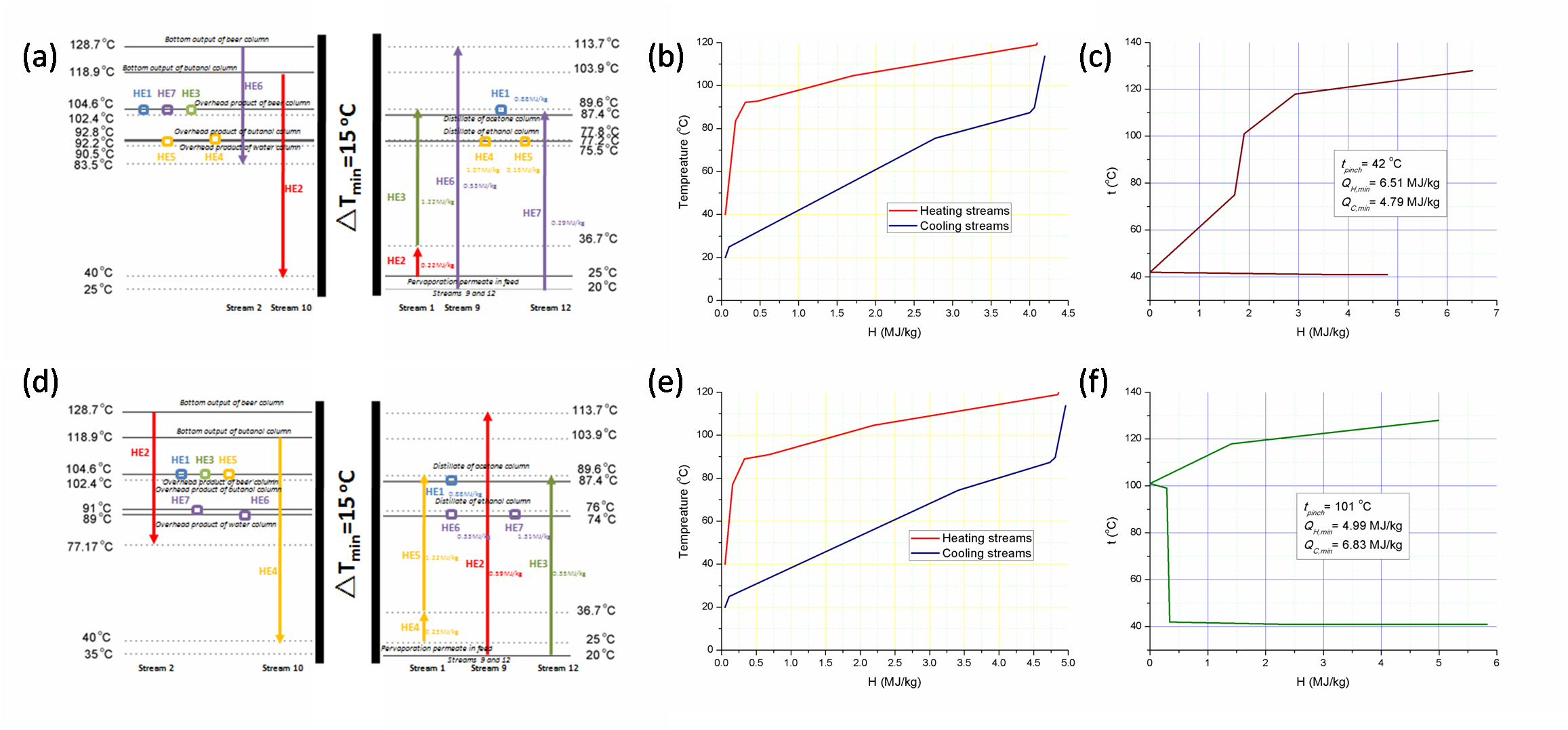


Table S1 Comparison of streams and flow rates of the TCD (scenario 1) and E-TCD (scenario 2) sequences based on atmospheric distillations.

| No. | Stream | Acetone (wt.%) | | Butanol (wt.%) | | Ethanol (wt.%) | | Water (wt.%) | | Flow rate (kg/h) | |
| --- | --- | --- | --- | --- | --- | --- | --- | --- | --- | --- | --- |
|  |  | Scenario 1 | Scenario 2 | Scenario 1 | Scenario 2 | Scenario 1 | Scenario 2 | Scenario 1 | Scenario 2 | Scenario 1 | Scenario 2 |
| 1 | Pervaporation permeate | 11.30 | 11.30 | 18.67 | 18.67 | 1.74 | 1.74 | 68.29 | 68.29 | 1025 | 1025 |
| 2 | Bottom output of beer column | 0 | 0 | 0 | 0 | 0 | 0 | 100 | 100 | 525.1 | 525.1 |
| 3 | Distillate of beer column | 23.16 | 23.16 | 38.28 | 38.28 | 3.56 | 3.56 | 34.99 | 34.99 | 499.9 | 499.9 |
| 4 | Distillate of acetone column | 99.7 | 99.7 | 0 | 0 | 0 | 0 | 0.3 | 0.3 | 116.1 | 116.1 |
| 5 | Bottom output of acetone column | 0 | 0 | 49.87 | 49.87 | 4.61 | 4.61 | 45.52 | 45.52 | 383.8 | 383.8 |
| 6 | Distillate of ethanol column | 0 | 0 | 0 | 0 | 95 | 95 | 5 | 5 | 18.62 | 18.62 |
| 7 | Bottom output of ethanol column | 0 | 0 | 52.41 | 52.41 | 0 | 2.42 | 47.59 | 45.16 | 365.2 | 605.4 |
| 8 | Distillate of butanol column | 0 | 0 | 34 | 31.65 | Trace | 4.82 | 66 | 63.54 | 160 | 190 |
| 9 | Organic phase of decanter | 0 | 0 | 85 | 81.66 | Trace | 2.98 | 15 | 15.36 | 351.5 | 381.4 |
| 10 | Bottom output of butanol column | 0 | 0 | 100 | 100 | Trace | 0 | 0 | 0 | 191.5 | 191.5 |
| 11 | Distillate of water column | 0 | 0 | 34 | 11.69 | Trace | 6.60 | 66 | 81.71 | 15 | 50 |
| 12 | Aqueous phase of decanter | 0 | 0 | 6 | 4.6 | 0 | 1.47 | 94 | 93.92 | 188.7 | 224 |
| 13 | Bottom output of water column | 0 | 0 | 0 | 0 | 0 | 0 | 100 | 100 | 173.7 | 173.7 |

Table S2 Comparison of streams and flow rates of the TCD (scenario 3) and E-TCD (scenario 4) sequences based on VDP.

| No. | Stream | Acetone (wt.%) | | Butanol (wt.%) | | Ethanol (wt.%) | | Water (wt.%) | | Flow rate (kg/h) | |
| --- | --- | --- | --- | --- | --- | --- | --- | --- | --- | --- | --- |
|  |  | Scenario 3 | Scenario 4 | Scenario 3 | Scenario 4 | Scenario 3 | Scenario 4 | Scenario 3 | Scenario 4 | Scenario 3 | Scenario 4 |
| 1 | Pervaporation permeate | 11.30 | 11.30 | 18.67 | 18.67 | 1.74 | 1.74 | 68.29 | 68.29 | 1025 | 1025 |
| 2 | Bottom output of beer column | 0 | 0 | 0 | 0 | 0 | 0 | 100 | 100 | 525.1 | 525.1 |
| 3 | Distillate of beer column | 23.16 | 23.16 | 38.28 | 38.28 | 3.56 | 3.56 | 35.00 | 35.00 | 499.9 | 499.9 |
| 4 | Distillate of acetone column | 99.7 | 99.7 | 0 | 0 | 0.17 | 0.17 | 0.13 | 0.13 | 116.1 | 116.1 |
| 5 | Bottom output of acetone column | 0 | 0 | 49.86 | 49.86 | 4.59 | 4.59 | 45.55 | 45.55 | 383.8 | 383.8 |
| 6 | Distillate of ethanol column | 0 | 0 | 0 | 0 | 95 | 95 | 5 | 5 | 18.62 | 18.62 |
| 7 | Bottom output of ethanol column | 0 | 0 | 52.39 | 53.58 | 0 | 3.08 | 47.61 | 43.34 | 365.2 | 625.3 |
| 8 | Distillate of butanol column | 0 | 0 | 34 | 62.36 | Trace | 7.3 | 66 | 30.34 | 160 | 200 |
| 9 | Organic phase of decanter | 0 | 0 | 85 | 80.76 | Trace | 3.73 | 15 | 15.5 | 351.5 | 391.4 |
| 10 | Bottom output of butanol column | 0 | 0 | 100 | 100 | Trace | 0 | 0 | 0 | 191.5 | 191.4 |
| 11 | Distillate of water column | 0 | 0 | 32 | 15.76 | Trace | 10.83 | 68 | 73.41 | 15 | 60 |
| 12 | Aqueous phase of decanter | 0 | 0 | 6 | 4.64 | 0 | 1.85 | 94 | 93.51 | 188.9 | 233.9 |
| 13 | Bottom output of water column | 0 | 0 | 0 | 0 | 0 | 0 | 100 | 100 | 173.7 | 173.9 |
